# Supplementary material for: Last-come, best served? Mosquito biting order and Plasmodium transmission
Source: Proc Biol Sci. 2020 Nov 25;287(1939):20202615. doi: 10.1098/rspb.2020.2615 (PMC7739503; doi:10.1098/rspb.2020.2615)
Supplement: Last-come, best served? Mosquito biting order and Plasmodium transmission: Supplementary materials [file rspb20202615supp1.pdf]

Last-come, best served? Mosquito biting order and *Plasmodium* transmission

J. ISAÏA[1], A. RIVERO[2,3], O. GLAIZOT[1,4], P. CHRISTE[1] & R. PIGEAULT[1,5]*

[1] Department of Ecology and Evolution, CH-1015 Lausanne, Switzerland

[2] MIVEGEC (UMR CNRS 5290), Montpellier, France

[3] CREES (Centre de Recherche en Ecologie et Evolution de la Santé), Montpellier, France

[4] Musée Cantonal de Zoologie, Lausanne, Switzerland

[5] Laboratoire EBI, Equipe EES, UMR CNRS 7267, Poitiers, France

*Corresponding author: romain.pigeault@unil.ch

Supplementary information

Table S1: Birds parasitaemia & gametocytaemia

Table S2: Description of statistical models

Table S1: Parasitaemia and gametocytaemia of birds used in the experiments. Exposed: birds exposed to mosquito bites, Unexposed: birds unexposed to mosquito bites.

Experiment	Bird_Ring_number	Status	Hour	Parasitaemia	Gametocytaemia
1_1	CH3430_1730_21	Exposed	6PM	2.20	0.09
			9PM	2.69	0.13
	CH3421_1727_04	Exposed	6PM	0.32	0.01
			9PM	0.38	0.01
	CH3421_1727_14	Unexposed	6PM	0.04	0.00
			9PM	0.08	0.01
	CH3421_1727_02	Unexposed	6PM	3.52	0.12
			9PM	3.44	0.11
	CH3421_1727_01	Unexposed	6PM	3.14	0.12
			9PM	2.73	0.04
	CH1648_1730_146	Unexposed	6PM	0.21	0.00
			9PM	0.05	0.02
	FOIB15_118x_29	Unexposed	6PM	0.71	0.09
			9PM	0.93	0.04
	CH1829_1730_80	Unexposed	6PM	17.22	0.84
			9PM	16.66	0.92
	CH3041_1730_21	Exposed	6PM	4.37	0.14
			9PM	6.03	0.13
	CH7706_1430_84	Exposed	6PM	14.97	1.81
			9PM	16.11	1.90
	CH6802_1630_3	Exposed	6PM	6.36	0.15
			9PM	5.74	0.22
	E7_15_1273_5	Exposed	6PM	12.05	0.81
			9PM	12.20	0.63
	CH3836_1730_25	Exposed	6PM	0.93	0.02
			9PM	0.75	0.02
	CH6802_1730_42	Exposed	6PM	2.88	0.17
			9PM	2.56	0.15
1_2	FOIB17_02UT_210	Exposed	6PM	0.02	0.00
			9PM	0.01	0.00
	FOIX18_50BM_24	Exposed	6PM	0.01	0.00
			9PM	0.01	0.00
	Bleu_Jaune	Unexposed	6PM	2.42	0.46
			9PM	3.35	0.35
	FOIX18_P848_43	Unexposed	6PM	0.01	0.00
			9PM	0.09	0.01
	FOIB17_31NM_501	Exposed	6PM	0.03	0.00
			9PM	0.02	0.00

2	1705_bleu	Unexposed	6PM	2.19	0.20
			9PM	2.68	0.27
	1718_rose	Exposed	6PM	1.76	0.10
			9PM	2.10	0.12
	CH1578_1727_7	Exposed	6PM	0.42	0.03
			9PM	0.56	0.01
	CH3430_1730_24	Unexposed	6PM	0.23	0.02
			9PM	0.21	0.02
	CH5361_1730_60	Exposed	6PM	1.27	0.09
			9PM	0.88	0.09
	DKB_0141_25_1730_86	Unexposed	6PM	1.27	0.05
			9PM	1.31	0.09
	DKB_0432_04_1730_123	Unexposed	6PM	1.68	0.28
			9PM	1.98	0.16
	Rouge/Violet	Exposed	6PM	2.63	0.33
			9PM	4.17	0.25
3	CH3445_1930_404	Exposed	6PM	6.02	0.31
			9PM	NA	NA
	CH3445_1930_351	Exposed	6PM	1.40	0.11
			9PM	NA	NA

Table S2: Description of statistical models presented in the main text. "N" gives the number of mosquitoes or birds included in each analysis. "Maximal Model" includes the complete set of explanatory variables. "Minimal model" gives the model containing only the significant variables and their interactions. Square brackets indicate variables fitted as random factors. Curly brackets indicate the error structure used (n: normal errors, b: binomial errors, nb: negative binomial errors).

Variable of interest	Resp. variable	Model Nb.	N	Maximal model	Minimal model	Rsubroutine [err struct.]
**Experiment 1_block 1**						
Blood meal rate	BM	1	800	Batch + (1/Bird)	1 + (1/Bird)	glmer[b]
Haematin	Hm	2	130	Batch + (1/Bird)	1 + (1/Bird)	lmer[n]
Infection prevalence	Prev	3	126	Batch * Hm + (1/Bird)	1 + (1/Bird)	glmer[b]
Oocyst burden	Oo	4	85	Batch * Hm + (1/Bird)	Batch + (1/Bird)	glmer[nb]
Oocyst burden*	Oo	5	78	Batch * Hm + (1/Bird)	Batch + (1/Bird)	glmer[nb]
Parasitaemia in birds	Par	6	14	Time * status + (1/Bird)	1 + (1/Bird)	lmer[n]
Gametocytaemia in birds	Gam	7	14	Time * status + (1/Bird)	1 + (1/Bird)	lmer[n]
**Experiment 1_block 2**						
Blood meal rate	BM	8	300	Batch + (1/Bird)	1 + (1/Bird)	glmer[b]
Haematin	Hm	9	161	Batch + (1/Bird)	1 + (1/Bird)	lmer[n]
Infection prevalence	Prev	10	161	Batch * Hm + (1/Bird)	1 + (1/Bird)	glmer[b]
Oocyst burden	Oo	11	104	Batch * Hm + (1/Bird)	Batch + Hm + (1/Bird)	glmer[nb]
Oocyst burden*	Oo	12	98	Batch * Hm + (1/Bird)	Batch + Hm + (1/Bird)	glmer[nb]
Parasitaemia in birds	Par	13	5	Time * status + (1/Bird)	1 + (1/Bird)	lmer[n]
Gametocytaemia in birds	Gam	14	5	Time * status + (1/Bird)	1 + (1/Bird)	lmer[n]

**Experiment 2**						
Haematin	Hm	15	67	Biting_Order + (1/Bird)	1 + (1/Bird)	lmer[n]
Infection prevalence	Prev	16	67	Biting_Order * Hm + (1/Bird)	1 + (1/Bird)	glmer[b]
Oocyst burden	Oo	17	66	Biting_Order * Hm + (1/Bird)	Biting_order + hm + (1/Bird)	glmer[nb]
Parasitaemia in birds	Par	18	8	Time * status + (1/Bird)	1 + (1/Bird)	lmer[n]
Gametocytaemia in birds	Gam	19	8	Time * status + (1/Bird)	1 + (1/Bird)	lmer[n]
**Experiment 3**						
Blood meal parasitaemia	RQ	20	44	Biting_Order + (1/Bird)	1 + (1/Bird)	lmer[n]
Haematin	Hm	21	47	Biting_Order + (1/Bird)	1 + (1/Bird)	lmer[n]
Infection prevalence	Prev	22	47	Biting_Order + (1/Bird)	1 + (1/Bird)	glmer[b]
Oocyst burden	Oo	23	46	Biting_Order * Hm + (1/Bird)	Biting_order + (1/Bird)	glmer[nb]

Variables: “Batch” = group of mosquitoes left 45 minutes in contact with birds (batch 1 ($T0_{min}$), batch 2 ($T45_{min}$), batch 3 ($T90_{min}$), batch 4 ($T135_{min}$), “Biting_Order” = mosquito biting order, “Time” = 6.00 PM or 9.00 PM, “status” = Bird exposed or not to mosquito bites. * Analysis without outliers.
